# Inhibition of USP14 enhances the sensitivity of breast cancer to enzalutamide

**DOI:** 10.1186/s13046-019-1227-7

**Published:** 2019-05-24

**Authors:** Xiaohong Xia, Chuyi Huang, Yuning Liao, Yuan Liu, Jinchan He, Zhiqiang Guo, Lili Jiang, Xuejun Wang, Jinbao Liu, Hongbiao Huang

**Affiliations:** 1 Affiliated Cancer Hospital and institute of Guangzhou Medical University; Key Laboratory of Protein Modification and Degradation; State Key Laboratory of Respiratory Disease, School of Basic Medical Sciences, Guangzhou Medical University, Guangzhou, 510095 Guangdong China; 2 Division of Basic Biomedical Sciences, Sanford School of Medicine of the University of South Dakota, Vermillion, SD 57069 USA

**Keywords:** Breast cancer, Enzalutamide, USP14, Androgen receptor, Combination treatment

## Abstract

**Background:**

Androgen receptor (AR) is expressed in approximately 70% of breast tumors. Recent studies increasingly support AR as a potential therapeutic target of AR-positive breast cancer. We have previously reported that deubiquitinase USP14 stabilizes AR proteins by deubiquitination and USP14 inhibition results in inhibition of cell growth and tumor progression in AR-positive prostate cancer and breast cancer. The current study aims to explore the anticancer effect of a treatment combining AR antagonist enzalutamide with USP14 inhibition on breast cancer cells.

**Methods:**

The combining effects of enzalutamide and USP14 inhibition on breast cancer cell proliferation and apoptosis and associated cell signaling were evaluated in vitro and in vivo.

**Results:**

USP14 inhibition via administration of IU1 or USP14-specific siRNA/shRNA enhanced cell growth inhibition and apoptosis induction by enzalutamide in breast cancer cell lines in vitro and in vivo. Additionally, the combination of enzalutamide with USP14 inhibition/knockdown induced significant downregulation of AR proteins and suppression of AR-related signaling pathways, including Wnt/β-catenin and PI3K/AKT pathways. Moreover, AKT inhibition via MK2206 increased the antiproliferative and proapoptotic effects of enzalutamide+IU1 combined treatment.

**Conclusion:**

Collectively, our data suggest that USP14 inhibition in combination with enzalutamide represents a potentially new therapeutic strategy for breast cancer.

**Electronic supplementary material:**

The online version of this article (10.1186/s13046-019-1227-7) contains supplementary material, which is available to authorized users.

## Background

Breast cancer (BCa) is the most common cancer in women worldwide. According to the United States Cancer Statistic, 123 new cases of breast cancer were diagnosed in 100,000 females every year and it has also been estimated that approximately 252,710 cases were found in 2017 in the U.S. The 5-year survival rate of metastatic breast cancer could only achieve 26%, even though some advanced treatments were adopted in the past 20 years [1]. For females, breast cancer is the fifth cause of death and in 2017 roughly 40,610 patients with breast cancer died in the U.S. [2]. Breast cancer is heterogeneous and exists different subtypes with variable outcome and time course. Breast cancer is classified further into four distinctive subtypes depending on the expression of estrogen receptor (ER), human epidermal growth factor receptor 2 (HER2) and progesterone receptor (PR) [3, 4]. Hormonal therapies aiming at ER, PR and HER2 have long been established and shown significant progress in treating patients with breast cancer [5, 6]. However, an incisive treatment for breast cancer to increase overall survival is deficient. Measures targeting new molecular pathways, such as the use of trastuzumab, tamoxifen and lapatinib to target HER2 and ER, have become important alternative therapies to traditional medications. To date, androgen receptor (AR) has emerged as a promising new therapeutic target in breast cancer therapy [7–9].

The AR makes a contribution to the progression and development of breast cancer and is expressed in all stages [10]. Approximately 77% patients with breast cancer were estimated to be AR positive [11]. Enzalutamide is considered as a potent AR signaling inhibitor approved for the therapy in men with metastatic castration-resistant prostate cancer [12]. Through competitively binding to AR, enzalutamide inhibits nuclear translocation of AR, androgen-mediated receptor activation, and the binding of AR to chromatin, resulting in inhibition of AR signaling and thereby leading to growth inhibition of prostate cancer cells, induction of apoptosis of prostate cancer cells and tumor regression in preclinical trials [13–15]. Moreover, it has been demonstrated that patients with breast cancer that express the androgen receptor tolerate enzalutamide well and benefit from enzalutamide treatment, suggesting enzalutamide has a significant antitumor effect and safety in AR positive breast cancer [16].

There are roughly 100 deubiquitinating enzymes (DUBs) in the ubiquitin-proteasome system [17]. Only three DUBs, including USP14, Rpn11, UCHL5, are present in mammalian 19S regulatory particles [18]. UCHL5 and USP14 play attractive and versatile roles, given their reversibility in association with the 19S proteasome [19]. USP14 is overexpressed in the most cancers and its deubiquitinating activity is activated by the proteasome. USP14 could reduce the anchoring time of ubiquitin conjugates with the proteasome and induce deubiquitination of targeted proteins to stabilize the substrate protein [20]. We have reported that USP14 mediates deubiquitination and stabilization of the AR in prostate and breast cancer cells [21, 22]. USP14 inhibition induced cell cycle arrest and apoptosis and overexpression of AR abrogated significantly the antiproliferative effect induced by USP14 siRNA in AR^+^ breast cancer cells [22].

In the present study, we sought to explore whether both inhibition of AR signal and degradation of AR protein would synergistically inhibit the growth and progression of breast cancer cells. Thus, we tested the combination effect of an AR antagonist (enzalutamide) and USP14 inhibitor (IU1)/siRNA in breast cancer cells. Our results provide strong experimental basis for this combination to become a rational and new therapeutic strategy for AR positive breast cancer.

## Methods

### Materials

IU1 (S7134), enzalutamide (S1250) and MK2206 (S1078) were obtained from Selleckchem (Houston, TX, USA). DMSO was used to dissolve these inhibitors and the inhibitors were stored at − 20 °C. USP14 (sc-76,817) siRNA was obtained from Santa Cruz Biotechnology (Santa Cruz, CA, USA). MTS (catalog no. G111) was obtained through Promega Corporation (Madison, WI, USA). Annexin V-FITC/PI apoptosis detection kits of Keygen Company (Nanjing, China)(KGA107) were purchased. Cell Lysis Buffer (#9803) was from Cell Signaling Technology (MA, USA) and stored at − 20 °C. Anti-GAPDH (MB001) was obtained from Bioworld Technology (St.Louis Park, MN, USA). The other antibodies were from Cell Signaling Technology (MA, USA): anti-PARP (#9542), anti-caspase 3 (#9668), anti-cleaved caspase 3 (#9661), anti-caspase 8 (#9746), anti-cleaved caspase 8 (#9496), anti-cleaved caspase 9 (#9501), anti-Bax (#5023), anti-CDK4(#12790), anti-P27(#3686), anti-caspase 9 (#9508), anti-CDK2 (#2546), anti-CyclinD1(#2922), anti-total-AKT (#9272), anti-AR (#5153), anti-USP14 (#11931), anti-Bcl-2 (#15071), anti-GSK3β (#12456), anti-p-GSK3β (Ser9)(#9323), anti-phospho-AKT (Ser473)(#4060), anti-β-catenin (#8480), anti-EGFR (#4267), anti-IGF1R (#9750).

### Cell lines and culture conditions

Human breast cell lines, including MDA-MB453, MCF-7, MDA-MB231, HCC1937 and MDA-MB468, were from American Type Culture Collection (Manassas, VA, USA). HyClone DMEM, 10% fetal bovine serum (FBS) was prepared to culture cells and cells were maintained at 37 °C and 5% CO2.

### Cell viability assay

As we previously reported, MTS was used to cell growth inhibition [23, 24]. In brief, exponentially growing MDA-MB453, MCF-7, MDA-MB231, HCC1937 and MDA-MB468 cells were digested and suspended at HyClone DMEM medium with 10% FBS. Then cells randomly were plated onto the 96-well plates with a volume of 100ul cell suspensions. After incubation overnight, the cells were treated with IU1/USP14 siRNA, enzalutamide or the combination of the two treatment for 72 h. 20 μl MTS was directly added to well and cells were incubated for an additional 3 h. The absorbance of optical density at wavelength of 490 nm with microplate reader (Sunrise, Tecan) was read.

### Colony formation assay

Clonogenic assay was performed as previously described [25]. MDA-MB453, MCF-7, MDA-MB231, HCC1937 and MDA-MB468 cells were exposed to IU1 and/or enzalutamide for 48 h. Then cells were digested and suspended in the 6-well plates containing 30% agarose supplemented with 10% FBS HyClone DMEM. The cells were cultured in an atmosphere of 5% CO2 at 37 °C for 10–14 days and washed with 4 °C PBS. 4% polyformaldehyde was added into 6-well plates to fix cells for 15 min and then crystal violet solution was diluted to 1% and used to stain cell for 5 min. Colonies > 60 μm were counted and the experiments were done in triplicate.

### EdU staining

The cell proliferation was detected with Cell-Light™ EdU Apollo 567 In Vitro Kit (Cat number: C10310–1, RiboBio, Guangzhou, China). Exponentially growing cells were digested and seeded at chamber slide overnight. The cells were exposed to IU1, enzalutamide or the combination for 48 h. 50 μmol/L Edu were added into chamber slide at 37 °C. After incubation for 2 h, the cells were washed with PBS twice and fixed with 4% paraformaldehyde for 30 min. Then 2 mg/ml glycine were used to incubate the cells for 5 min and washed with PBS. 0.5% Triton X-100 was used to incubate cell for 10 min and cell was washed with PBS once. Apollo reaction cocktail containing annexing agent buffer, fluorochrome, catalytic agent and Apollo reaction buffer was already to incubate cells. After 30 min, 0.5% Triton X-100 was used to wash cell for 10 min twice. Fluoroshield mounting medium with DAPI was added for DNA staining in dark. Image were captured using an Olympus microscope from three independent repeated experiments.

### Flow cytometry analysis of cell cycle and apoptosis

Apoptosis assay was performed according to previous description [26]. MDA-MB453 and MCF-7 cells treated IU1 and/or enzalutamide for indicated time were harvested and washed with PBS twice. Then 500 μl binding buffer was already to suspend cells. Annexin V-FITC/ PI was added into tube and incubated for 15 min in dark, followed were detected by flow cytometry.

For cell cycle assay, cells were exposed to either IU1, enzalutamide or the combination of both IU1 and enzalutamide for indicated time. Cells were digested by trypsinization and then washed with cold PBS twice. Then cells were suspended and fixed with 500 μl PBS and 2 ml 70% ethanol overnight. Lastly, PBS was used to wash cells thrice and cells were incubated with PI, RNase A and 0.2% Triton X-100 complexes for 30 min at 4 °C in dark.

### SiRNA transfection

As previously described [27], MDA-MB453 cells were harvested and seeded in plates or dishes overnight. 500 μl RPMI opti-MEM and 5 μl lipofectamine RNAimax (Invitrogen) reagent mixtures were prepared. SiRNA targeting human USP14 or siRNA with non-specific sequences were added into the mixtures. The cell was incubated with mixtures. After transfection for 6 h, 10% FBS HyClone DMEM was replaced and incubated for additional 42 h.

### Lentivirus USP14 shRNA transfection

Lentiviruses (pLent-4in1shRNA-GFP) expressing control shRNA or human USP14-speicfic shRNA (NM-005151) were purchased from VigeneBio (Shandong, China). Cultured cells were digested and plated into 6 cm dishes. After 24 h, polybrene (Santa Cruz, CA, USA) and lentivirus mixtures were dissolved in medium and added into each well. When cells were incubated overnight, supernatant was replaced with fresh medium and cultured for 48 h. In order to select stably-transfected cells, puromycin (Santa Cruz, CA, USA) was used at the concentration of 2 μg/ml to perform the selection.

### Western blot

This assay was performed as we previously reported [28]. Breast cancer cell was exposed to the indicated treatment and the total proteins were extracted using cell lysis buffer. Collected proteins were quantitated using BCA protein assay kit. The protein samples were prepared and then separated by SDS-PAGE. Then the fractionated proteins were transferred to PVDF membranes. Lastly, to block bolts, the membranes were incubated with 5% defatted milk powder for one hour and PBS-T were used to wash the membranes for three times for 5 min. Importantly, the membranes were incubated with primary antibodies at 4 °C overnight and washed with PBS-T for 6 min thrice. Secondary antibodies were used to incubate the membranes. After 1 h, the membranes were washed with PBS-T for 6 min for thrice. Lastly, the ECL detection reagents were used to react with bounded secondary antibodies and exposed to X-ray films (Kodak, Japan).

### Immunofluorescence assay

Immunoflurescence assay was performed as we described previously [22]. MDA-MB453 cells were treated with IU1/USP14 siRNA, enzalutamide or the combination for indicated time. The medium was removed and PBS was used to wash. Then 4% paraformaldehyde for 15 min to fix the cells, followed by permeabilization with 0.1% Triton X-100 (Solarbio Life Science). After 10 min, cells were blocked with 5% BSA and then primary antibody diluted with 1% BSA was used to incubate overnight at 4°L. Lastly, the incubated of secondary Cy3-conjugated antibody was performed and fluoroshield mounting medium with DAPI (Abcam) was used. Image were captured using fluorescence microscope in three times.

### Luciferase reporter promoter assay

MDA-MB453 and MCF-7 cells were seeded into 24-well plates for 24 h. The mixture containing 500 μl RPMI opti-MEM, 5 μl iMAX and 1000 ng luciferase reporter was added into cells for 24 h. Then cells were treated with IU1, enzalutamide or USP14 siRNA for the indicated time. The activity of luciferase was measured using dual luciferase assays kit according to the manufacture’s instructions.

### Nude mouse xenograft model

The mice were purchased from Guangzhou University of Chinese Medicine and animal protocols were approved by the Institutional Animal Care and Use Committee of Guangzhou Medical University. The nude BALB/c mice (18–22 g, female) were housed in quarantine room for inspection for 2–3 days. Then mice were transferred to barrier facilities in the animal facility of Guangzhou Medical University. Water and food were available ad libitum. After then, the healthy mice were subcutaneously inoculated with MCF-7 cells or MCF-7 cells stably expressing USP14 shRNA or control shRNA in the left armpit. After one month, the inoculated mice were randomly divided into 4 × 4 groups and orally administered with IU1 (40 mg/kg/d) and/or enzalutamide (25 mg/kg/d) for 17 days. Tumor volumes were calculated and mouse body weight were measured every other day.

### Immunohistochemical staining

According to standard techniques as we previously reported [29, 30], fixed xenografts were embedded in paraffin and sectioned. The tumor sections were immunostained using MaxVision kits (Maixin Biol). Then the tissue samples were subjected to immunohistochemistry using AR, USP14,Ki67 and p-AKT. Each slide was added using 50 μl MaxVisionTM reagent and stained with 0.05% diaminobenzidine and 0.03% H2O2 in 50 mM Tris-HCl (pH 7.6). Hematoxylin was used to counterstain the slides. The primary antibodies were determined with DAB.

### TUNEL staining

Apoptosis cells of subcutaneous tumor in breast cancer were detected by terminal deoxynucleotidyl deoxyuridine triphosphate nick-end labeling (TUNEL) assay. The paraffin-embedded sections of breast tumor firstly baked at 60 °C for 30 min. Then the sections were dewaxed with xylene at 50 °C for 30 min followed by gradient alcohol and proteinase K for 20 min at 37 °C. The sections were washed with PBST for three times and soaked in 3% H_2_O_2_. TUNEL reaction mixture buffer was used to incubate with the sections for 1 h and then the sections were incubated with converter-POD for additional 30 min at 37 °C in dark. Finally, the sections were reacted with DAB for 3 min and the images were captured by a fluorescence microscope.

### Statistical methods

The data of all experiments were from three independent experiments that applicable and are presented as mean ± SD. Unpaired Student’s *t* test or one way ANOVA were used to determine statistical probabilities. Graph Pad Prism 5.0 software (GraphPad Software) was applied for statistical analysis and *P* value less than 0.05 was considered statistically significant.

## Results

### High expression of USP14 in breast cancer tissues and its correlation to AR expression

**Fig. 1:**
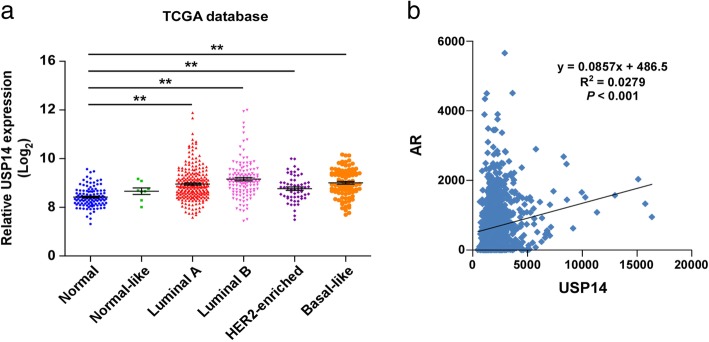
High expression of USP14 in breast cancer tissues and its correlation to AR expression. **a** Data of USP14 expression in breast cancer and normal tissues from the TCGA database were analyzed and presented. Each dot represents a patient sample (Normal, *n* = 113; Normal-like, *n* = 8; Luminal A, *n* = 231; Luminal B, *n* = 127; HER2-enriched, *n* = 58; Basal-like, *n* = 97). ** *P* < 0.01. **b** The correlation of USP14 expression with AR expression in AR-positive breast cancer tissues was detected by analyzing TCGA database (*n* = 1095)

The results from analyzing the TCGA database suggested that the mRNA expression of USP14 in all subtypes of Bca tissues was remarkably higher than in normal tissues (Fig. 1a). To explore the relationship between USP14 and AR, we analyzed the expression levels of USP14 in AR positive breast cancer. The results show a statistically significant positive correlation between USP14 expression and AR expression in breast cancer (Fig. 1b), suggesting that the increased USP14 expression might have resulted from elevated AR expression.

### Enzalutamide and USP14 inhibition synergistically inhibits the proliferation of breast cancer cells

**Fig. 2:**
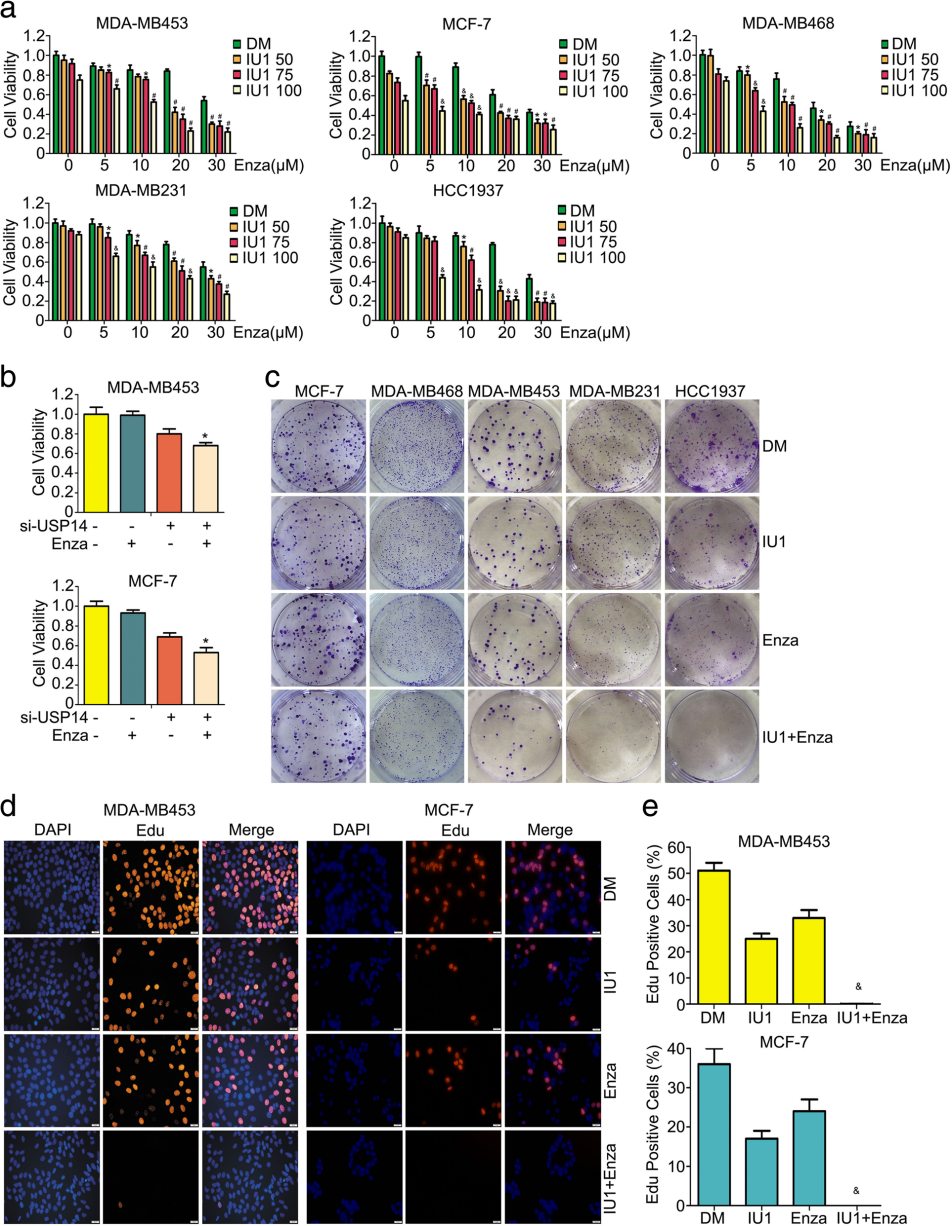
Enzalutamide and USP14 inhibition synergistically suppress the proliferation of breast cancer cells. **a** MCF-7, MDA-MB453, MDA-MB231, MDA-MB468 and HCC1937 cell lines were plated in 96-well plates and exposed to enzalutamide (Enza; 0, 5, 10, 20, 30 μM), USP14 inhibitor (IU1: 0, 50, 75, 100 μM), or the combination of enzalutamide +USP14 inhibitor for 72 h in triplicates. Cell viability was measured after adding 20 μl MTS for 2 h. *p < 0.05, ^#^*p* < 0.01, ^&^*p* < 0.001 vs. each treatment alone. DM: DMSO. **b** The indicated breast cancer cells in cultures were treated with USP14-speicfic siRNA (si-USP14, 30 nM), enzalutamide (20 μM), or both for 72 h in triplicates and then subject to the MTS assay. *p < 0.05 vs. each treatment alone. **c** The indicated 5 breast cancer cell lines were treated with enzalutamide (Enza, 20 μM), IU1 (75 μM), or the combination of the two agents for 48 h before they were used for the colony formation assay in which the treated cells were re-plated in 6-well plates cultured for approximately 10 days before processed for detection of the colonies. Representative images are shown. **d** and (**e**) Cell proliferative ability was detected using Edu staining in MDA-MB453 and MCF-7 cells treated with enzalutamide (Enza, 20 μM), IU1 (75 μM), or the combination of both (IU1 + Enza) for 48 h. Nuclei were stained with DAPI. The representative images (**d**) and pooled data (**e**) from three independent experiments are shown. Scale bar, 50 μm. ^&^p < 0.001 vs. each treatment alone

To assess the antiproliferative effects of enzalutamide in different doses, alone or in combination with USP14 specific inhibitor IU1 [31] on breast cancer cells, we used an MTS assay to test cell viability on a panel of 5 breast cancer cell lines. We found that either enzalutamide or IU1 alone induced cell growth inhibition in a concentration-dependent manner. Importantly, the combination of enzalutamide and IU1 showed a significantly greater inhibitory effect either agent alone (Fig. 2a). In our previous study, we have detected AR protein expression in all of the five breast cancer cell lines used here: MDA-MB453, MCF-7, MDA-MB468, MDA-MB231 and HCC1937; however, the highest AR protein expression was found in MDA-MB453 and MCF-7 cell lines [22]. Therefore, MDA-MB453 and MCF-7 cell lines were selected as the main targeted cells to test the effect of enzalutamide in combination with IU1. To corroborate that the enhancement effect of IU1 in the combined treatment is through USP14 inhibition, we also tested whether genetic inhibition of USP14 would yield similar effects using USP14 small interfering RNA (siRNA) to knock down USP14 expression in MDA-MB453 and MCF-7 cells. USP14 knockdown induced significant cell growth inhibition and increased enzalutamide-induced antiproliferation effect (Fig. 2b). Furthermore, overexpressing USP14 partly rescued cell growth inhibition induced by enzalutamide (Additional file 1: Figure S1e), suggesting that the combination induced cellular events dependent on USP14 status. Next, we further tested the long-term effect of enzalutamide, IU1, or a combination of both on the five breast cancer cell lines mentioned above using the colony formation assay. As shown in Fig. 2c, the colony forming ability of the cells treated with either enzalutamide or IU1 alone was decreased than that of the cells treated with vehicle control but, more remarkably, this decrease in colony formation was more pronounced in the cells treated with a combination of enzalutamide and IU1. Edu is a thymidine analog and can be incorporated into the replicating chromosomal DNA during the S phase of cell cycle, which is exploited for detection of DNA synthesis in the Edu labeling assay [32]. To further determine whether enzalutamide and IU1show synergy in the antiproliferative effect on breast cancer cells, we performed Edu labeling assay on MDA-MB453 and MCF-7 cells exposed to enzalutamide,IU1, or a combination of both. We found that the percentage of cells positively labeled with Edu in the group received the treatment combining enzalutamide and IU1 was drastically lower than that in the groups treated with either agent alone (Fig. 2d and e). These results compellingly demonstrate that enzalutamide and IU1 synergistically enhances each other’s antiproliferative effects in breast cancer cells.

### Induction of apoptosis by the co-treatment of enzalutamide and USP14 inhibition in breast cancer cells

**Fig. 3:**
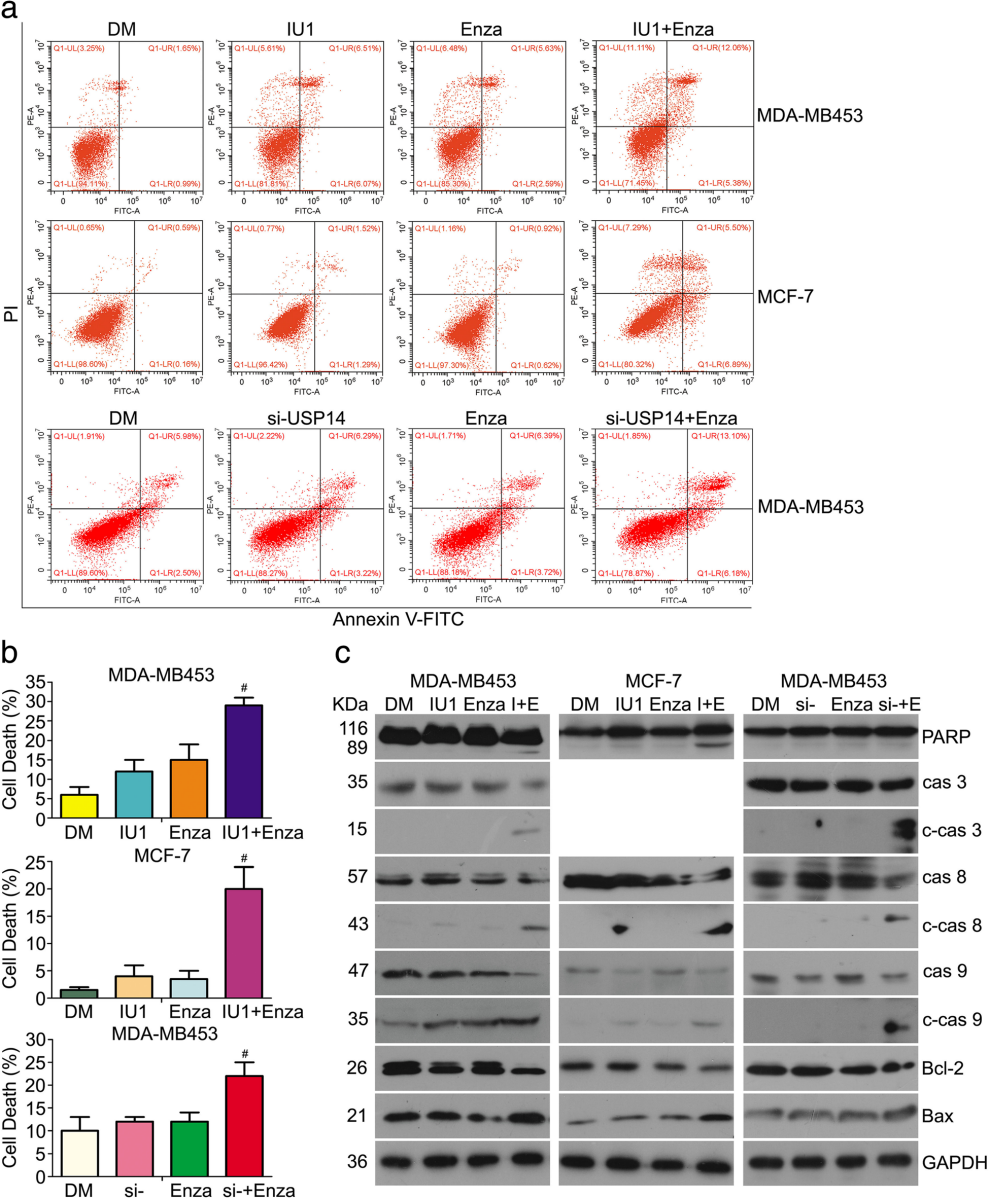
Induction of apoptosis by the co-treatment of enzalutamide and USP14 inhibition in breast cancer cells. MDA-MB453 and MCF-7 cells were treated with vehicle control (DM), enzalutamide (Enza, 20 μM), IU1 (75 μM), or the combination of both (Enza+IU1). Or MDA-MB453 cells were similarly exposed to enzalutamide (Enza or E; 20 μM), USP14 siRNA (si-; 50 nM), or the combination of both. At 48 h post-treatment, the cells were either processed for Annexin V-FITC/PI staining followed by flow cytometry (**a**) and (**b**) or harvested for western blot analyses for the indicated proteins in total cell lysates (**c**). GAPDH was used as a loading control. Representative histograms (**a**) and the percentage of cell death (**b)** derived from flow cytometry as well as the representative images of western blot (**c**) are shown. ^#^*p* < 0.01 vs. each treatment alone

To further explore the mechanism of the antiproliferative effect induced by enzalutamide in combination with IU1, we next investigated whether apoptosis was triggered and involved in cell growth inhibition. Induction of apoptosis in MDA-MB453 and MCF-7 cells after 48 h of treatment with enzalutamide, IU1, or a combination of the two was assessed with flow cytometric analyses of Annexin V-FITC/PI stained cells. We found that less than 10% of cells in the enzalutamide or IU1-treated groups underwent apoptosis but the treatment combining enzalutamide with IU1 increased the apoptotic cell percentage to more than 20% in both cell lines. Also, when siRNA-mediated USP14 knockdown was used to replace the IU1 treatment in MDA-MB453 cells, similar results were obtained (Fig. 3a and b). We further measured the expression level of proteins associated with apoptosis. Neither IU1 nor enzalutamide alone induced significant cleavage of the 113-kDa PARP to the 89-kDa fragment in MDA-MB453 and MCF-7 cells. However, the 89-kDa apoptotic fragment of PARP was remarkably induced in the combined treatment of enzalutamide and IU1. Importantly, apoptotic “executioners” including the cleaved caspase − 3, − 8, − 9 were increased in the co-treatment group, compared with the single agent treatment groups in MDA-MB453 and MCF-7 cells (MCF-7 is deficient of caspase-3), suggesting that caspase plays a critical role in apoptosis induced by co-treatment of enzalutamide and IU1. The BCL-2 family are known to regulate cancer cell survival and death and are closely related to the apoptotic pathway [33]; hence, we also measured the expression of Bax and Bcl-2, two main majors in the BCL-2 family using western blot analyses. We found that the anti-apoptotic protein Bcl-2 was decreased and pro-apoptotic protein Bax was increased by the treatments. More importantly, the downregulation of Bcl-2 and upregulation of Bax were more pronounced in the combined treatment group. USP14 siRNA in combination with enzalutamide induced similar results in MDA-MB453 cells (Fig. 3c). These results suggest that the antiproliferative effect induced by treatment combining enzalutamide with USP14 inhibition is associated with cell death.

### The treatment combining enzalutamide with USP14 inhibition arrests cell cycle progression

**Fig. 4:**
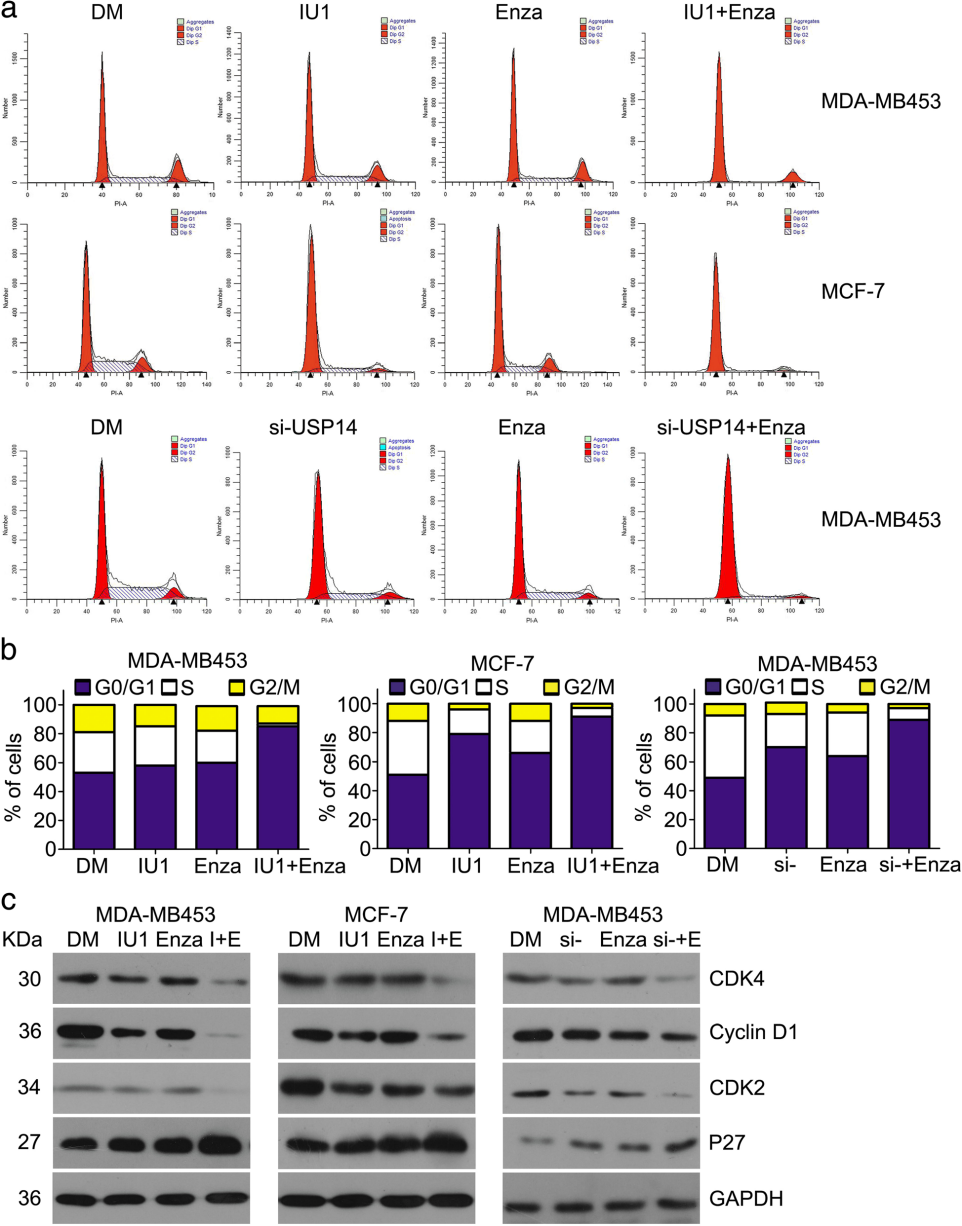
The treatment combining enzalutamide with USP14 inhibition arrests cell cycle progression MDA-MB453 and MCF-7 cells were treated with the indicated agents as described in Fig. 3 and then either processed for fluorescence-activated cell sorting analysis (FACS) to detect cell cycle distribution (**a**) and (**b**) or subject to total protein extraction for western blot analyses of the indicated cell cycle regulator proteins (**c**). GAPDH was used as a loading control. Representative images (**a**) and a summary of cell cycle distribution **b** derived from the FACS or representative images of the western blot analyses (**c**) are shown

The deregulation of cell cycle is linked to oncogenesis in various cancers [34]. To explore the mechanisms underlying the synergistic antiproliferation effect between enzalutamide and USP14 inhibition, we determined their effects on cell cycle progression using flow cytometry analysis and on the protein expression of key cell cycle regulators using western blot analyses. We found that both enzalutamide and IU1 or USP14 siRNA arrested cell cycle progression at the G0/G1 phase. Importantly, 90% or more of the cells subject to the treatment combining enzalutamide with IU1 or with USP14 siRNA were arrested at the G0/G1 phase, remarkably greater than that of the cells treated with enzalutamide or USP14 inhibition alone (Fig. 4a and b). Cyclin D1, CDK4, CDK2, and P27 are key regulators for the G1 to S phase transition. Our western blot analyses showed that the expression of CDK4, CDK2 and Cyclin D1, which promote cell cycle progression, were decreased and P27 which blocks cell cycle at G0/G1 phase was increased by treatment with enzalutamide, IU1, or USP14 siRNA. Furthermore, these changes were more remarkable in the cells treated with a combination of enzalutamide with IU1 or USP14 siRNA (Fig. 4c). These results are in agreement with the flow cytometry data, indicating that the treatment combining enzalutamide with USP14 inhibition induces cell cycle arrest at G0/G1 phase potentially; these findings also suggest that the cell cycle arrest is mediated by decreasing CDK4, CDK2, and Cyclin D1 and increasing P27 protein expression.

### The treatment of combining enzalutamide with and IU1 or USP14 knockdown induces the downregulation of AR and inhibits AR-related signaling pathways

**Fig. 5:**
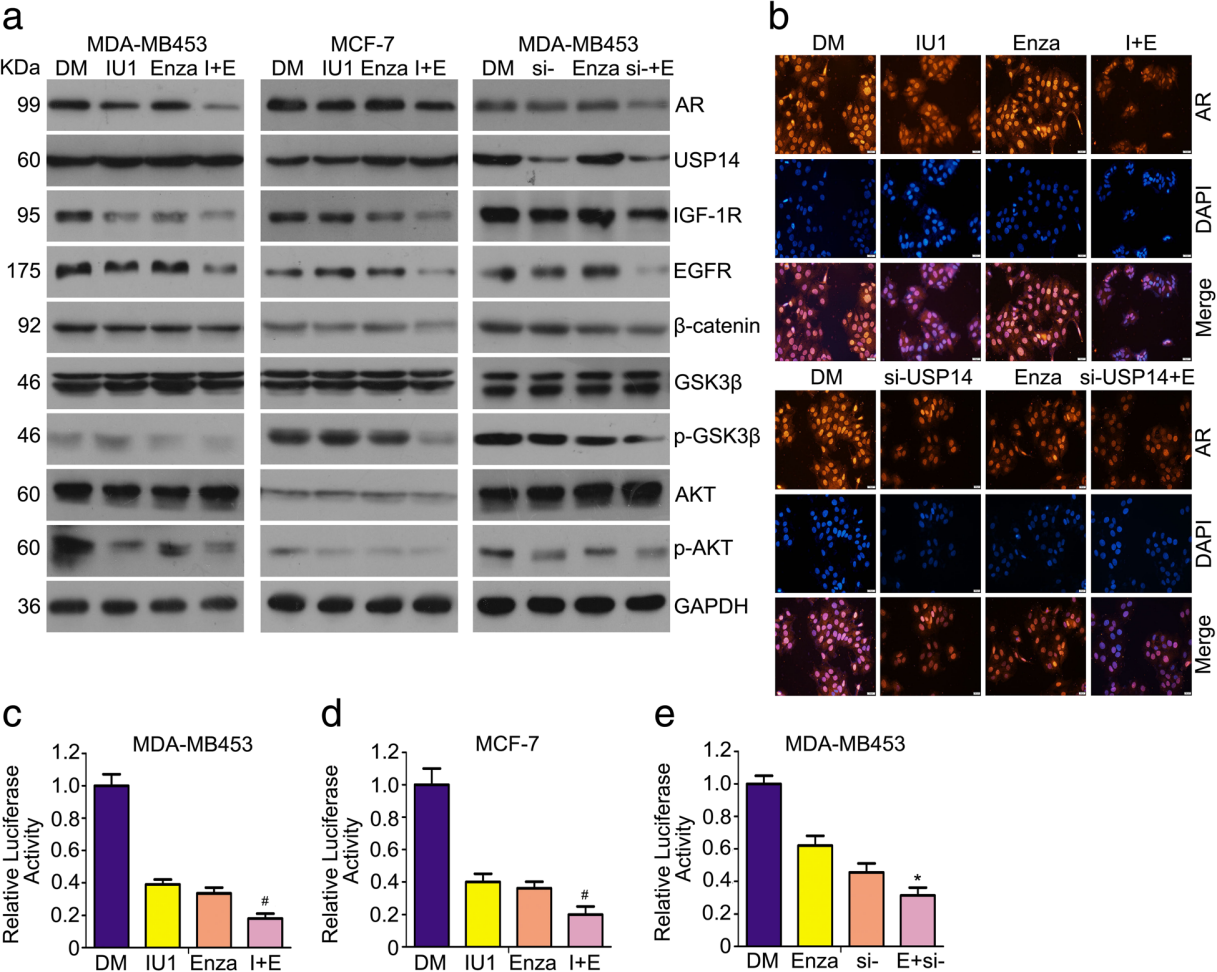
The treatment of combining enzalutamide with and IU1 or USP14 knockdown induces the downregulation of AR and inhibits AR-related signaling pathways. **a** MDA-MB453 and MCF-7 cells treated with the inidicated agents for 48 h as described in Fig. 3 were harvested for extraction of total proteins which were used for western blot analyses of the indicated proteins. **b** MDA-MB453 cells were treated with the indicated agents for 24 h as described in Fig. 3 and then fixed and processed for immunostaining for AR (red). Nuclei were counter-stained with DAPI (blue). Fluorescence microscopy was then used to record the expression and distribution of endogenous AR. The images shown are representative of three independent experiments. Scale bar, 50 μm. **c-e** MCF-7 and MDA-MB453 cells were transfected with luciferase reporter plasmid for 24 h. Then cells were treated with enzalutamide and USP14 inhibitor or siRNA. Protein lysates were collected, followed by dual-luciferase assay for luciferase activity. *p < 0.05, ^#^p < 0.01 vs. each treatment alone

The protein level of AR is significantly associated with disease outcome in breast cancer [35]. Importantly, AR may be considered as a target in the standard chemotherapeutic regimen for breast cancer. Next, we tested the effect of the treatment combining pharmacological or genetic inhibition of USP14 inhibition with enzalutamide on AR protein expression and signaling. Western blot analyses showed that enzalutamide promoted the downregulation of AR protein induced by IU1 or USP14 siRNA (Fig. 5a). The cross-talks exist between AR and other molecules that have been regarded as significant biological targets in clinical trials. Androgen receptor could regulate the expression of IGF-1 receptor (IGF-1R) via a non-genomic pathway [36]; for example, the re-expression of AR in M12 prostate cancer cells increased IGF-1R expression [37]. In addition, overexpression of AR results in an increase in the protein level of epidermal growth factor receptor (EGFR) [38]. We found that the treatment combining IU1 or USP14 siRNA with enzalutamide remarkably decreased the expression of IGF-1R and EGFR proteins (Fig. 5a). AR expression is associated with the PI3K signaling pathway [39]. The wnt/β-catenin are involved in AR signaling. The phosphorylation of GSK-3β by AKT mediates abnormal expression of β-catenin and β-catenin has impact on the transactivation of AR to drive the progression in cancer [40]. As shown in Fig. 5a, the combination of enzalutamide with USP14 inhibition more remarkably inhibited PI3K and wnt/β-catenin signaling pathways, compared with the single drug treatment. These findings suggest that the combination of USP14 inhibition with enzalutamide decreases AR protein expression and inhibits AR-related intracellular signaling pathways. To further explore the role of USP14 inhibition and enzalutamide on AR, we sought to determine whether they could interfere AR nuclear translocation by performing immunofluorescent staining assays to observe AR protein distribution and expression in MDA-MB453 cells. We found that the AR immunofluorescence in the cells received the co-treatment of enzalutamide with IU1 or with USP14 siRNA was markedly less intense than in those received the single agent treatment; however, the nuclear and cytoplasmic distribution of AR staining was comparable among different treatment groups (Fig. 5b), indicating that the combined treatment exerts its synergistic effect on suppressing AR signaling mainly through reducing AR protein levels not suppressing AR nuclear translocation in breast cancer cells. Additionally, we asked whether the combination of enzalutamide and USP14 inhibition alter AR-mediated transcription activity. The results of luciferase reporter assay reveal that AR-mediated transcription activity is inhibited by the co-treatment of enzalutamide and USP14 inhibition (Fig. 5c-e). Although quite a few studies try to show the role of AR in breast cancer cells, the function of AR in breast cancer is not totally clear. To further confirm the role of AR in AR positive breast cancer cells. We explored the effect of AR depletion in breast cancer cells. We found that cell viability, proliferation and colony forming ability were suppressed by AR knockdown. Moreover, AR silence induced cell apoptosis in MDA-MB453 cells. Significantly, the results showed that AR depletion enhances enzalutamide induced-antiproliferative effect (Fig. S1a-d). These results indicated that induction of growth inhibition in co-treatment of enzalutamide and USP14 depletion could be a consequence of blocking AR signaling.

### Synergistic growth inhibition by the co-treatment of enzalutamide and USP14 inhibition involves mechanisms more than suppressing AR-PI3K/AKT signaling

**Fig. 6:**
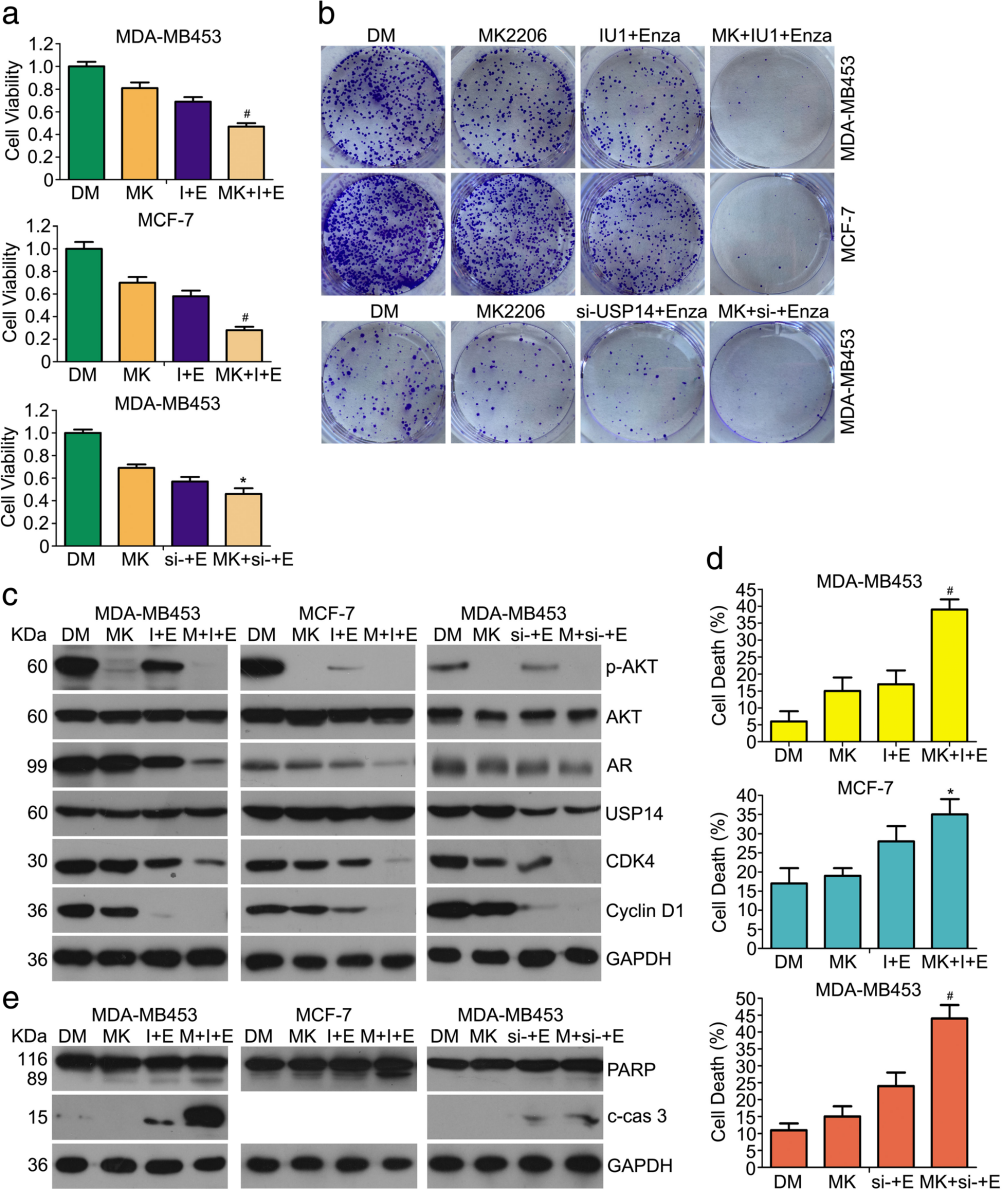
Synergistic growth inhibition by the co-treatment of enzalutamide and USP14 inhibition involves mechanisms more than suppressing AR-PI3K/AKT signaling. MDA-MB453 and MCF-7 cells exposed to MK2206 (MK, 5 μM), enzalutamide+IU1/USP14 siRNA (I + E or si − +E), the combination of the three agents (MK + I + E or MK + si − +E), or vehicle control DMSO (DM) for 48 h were used for the following assays. **a** Cell viability assay.*p < 0.05, ^#^p < 0.01 vs. other treatment group. **b** Colony formation assay performed as described in Fig. 2. Representative images are shown. **c** Representative images of Western blot analysis for the indicated proteins. GAPDH was probed as a loading control. **d** Prevalence of cell death detected with the same flow cytometry analysis as described in Fig. 2. *p < 0.05, ^#^p < 0.01 vs. other treatment group. **e** representative images of western blot analyses for PARP and cleaved caspase-3 in the total protein extracted from the treated cells

To better understand whether cell growth inhibition induced by the combination of USP14 inhibition and enzalutamide is associated with PI3K/AKT inhibition from the AR signaling suppression, the effect of AKT inhibitor (MK2206) on the cell survival and growth in MCF-7 and MDA-MB453 cells receiving the duo-treatment was examined. The MTS assays revealed that the molecular inhibitor of AKT increased the antiproliferation effect of enzalutamide+USP14 inhibition co-treatment (Fig. 6a). In addition to cell viability assay, we performed the colony formation assay and found that cell clonogenicity was decreased in the cells receiving MK2206 + enzalutamide+USP14 inhibition triple treatment, compared with enzalutamide+USP14 inhibition duo-treatment (Fig. 6b). The protein levels of CDK4 and Cyclin D1 were further decreased after adding MK2206 to the treatment of enzalutamide+IU1/USP14 siRNA. AKT inhibitor promoted the downregulation of AR protein induced by the combination of enzalutamide and IU1/USP14 knockdown and the cell cycle arrest was more remarkable in the triple treatment group (Fig. 6c). Moreover, the rates of cell death were significantly higher in the triple treatment group, compared with the enzalutamide+USP14 inhibition duo-treatment group (Fig. 6d). Inhibition of AKT exacerbated enzalutamide+IU1-induced the expression of cleaved caspase-3 and PARP (Fig. 6e). These results suggest that although suppression of PI3K/AKT signaling is involved in the synergic antiproliferation effect resulting from the synergistic suppression of AR signaling by enzalutamide and USP14 inhibition, other antiproliferative mechanisms from USP14 inhibition may also contribute to the synergy.

### Co-treatment of enzalutamide and USP14 inhibition suppresses the growth of breast cancer in vivo

**Fig. 7:**
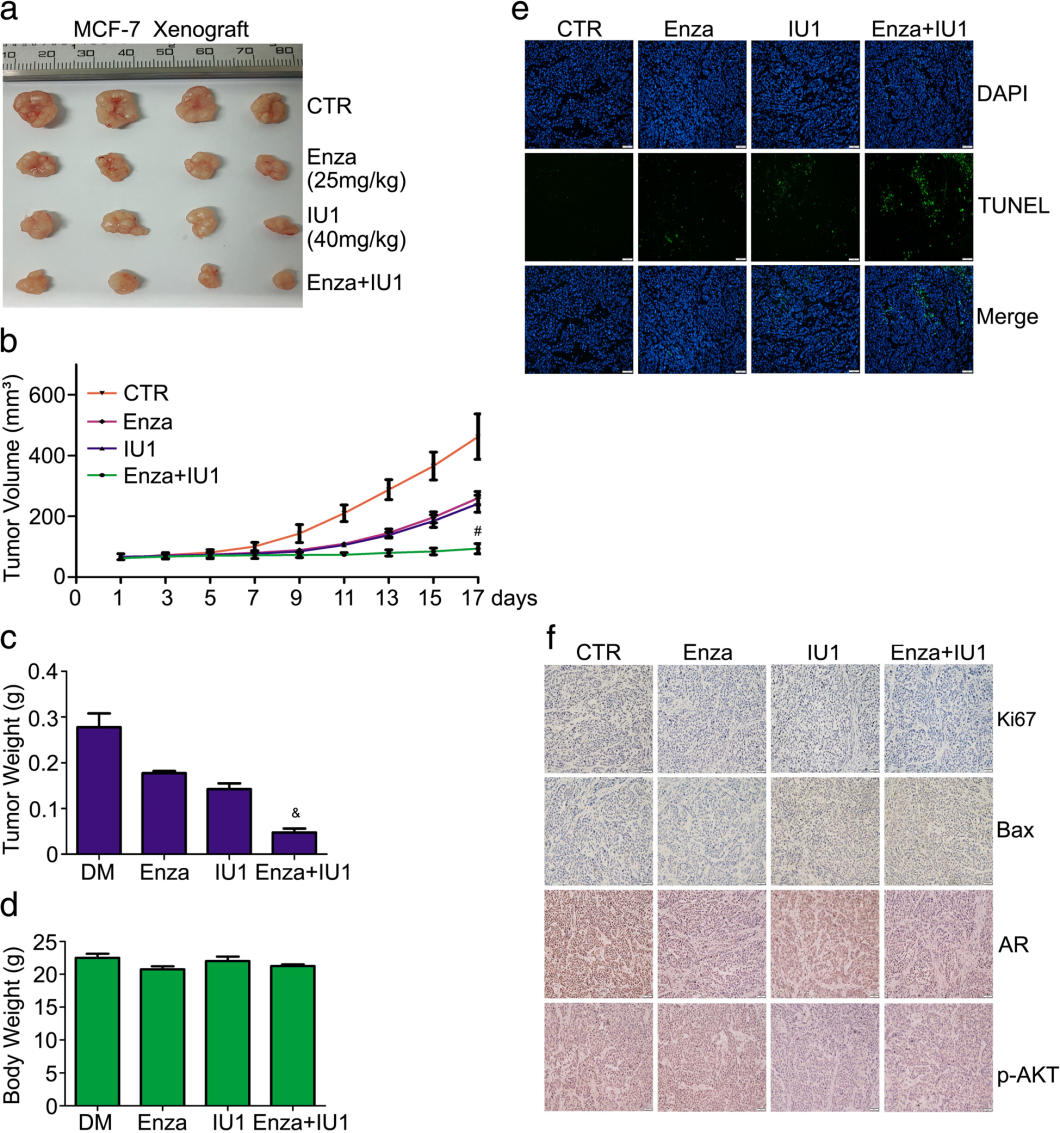
Co-treatment of enzalutamide and USP14 inhibition suppresses the growth of breast cancer in vivo. BALB/c nude mice subcutaneously injected with MCF-7 cells were treated with enzalutamide (25 mg/kg), IU1 (40 mg/kg), or enzalutamide+IU1 for 17 days. Tumor volume (**b**) and mouse body weight (**d**) were measured every other day. At the end of 17 days of treatment, the tumor xenograft was excised, imaged (**a**), weighed (**c**), and further processed for TUNEL staining (**e**). Scale bar, 20 μm. And immunohistochemistry staining for AR, p-AKT, Bax and Ki67 (**f**). Scale bar, 50 μm. ^#^p < 0.01, ^&^*p* < 0.001 vs. other treatment group

**Fig. 8:**
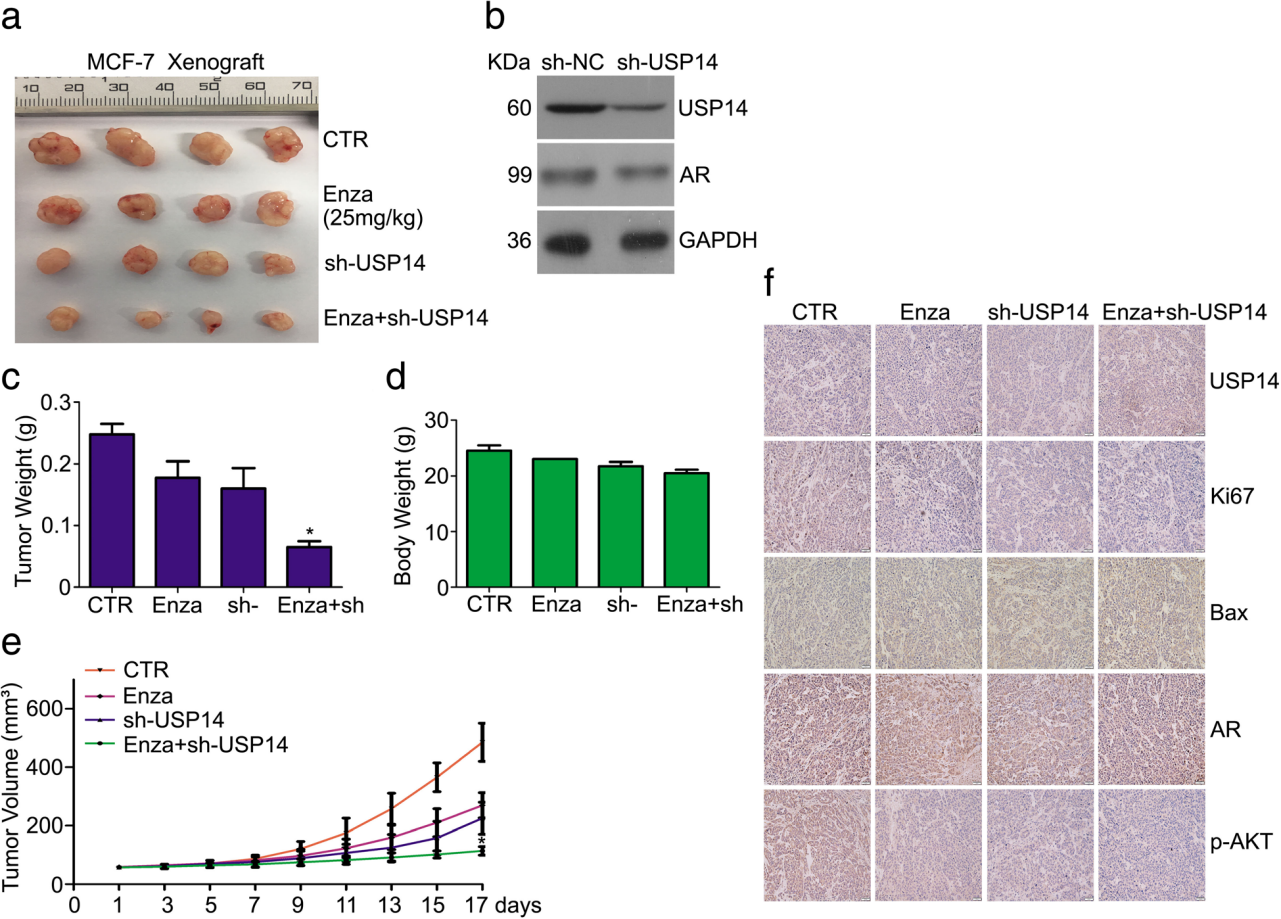
Co-treatment of enzalutamide and USP14 knockdown suppresses the growth of breast cancer in vivo. BALB/c nude mice, at one month after inoculation with MCF-7 cells stably expressing either human USP14-speicfic shRNA or control shRNA, were administrated orally with enzalutamide (25 mg/kg, daily) for 17 days. **a** Xenograft images, (**c**) tumor weight, (**d**) mouse body weight, (**e**) tumor volume were presented. *p < 0.05 vs. other treatment group. **b** Western blot analysis for AR and USP14 in the cancer cells before inoculation were performed. **f** Representative micrographs of immunohistochemistry staining for AR, USP14, p-AKT, Bax and Ki67 in explanted tumor tissues. Scale bar, 50 μm

Lastly, we tested the antitumor activity of the co-treatment of enzalutamide and IU1/USP14 knockdown in nude mice bearing tumor xenografts from MCF-7 breast cancer cells. Enzalutamide resulted in xenograft shrinkage and the similar result was obtained by IU1. Importantly, a synergic effect of enzalutamide in combination with IU1 on tumor growth inhibition was found upon co-administration of the two agents for 17 days (Fig. 7a-c); tumor volumes and tumor weight were remarkably declined in the combination therapy as compared with the enzalutamide or IU1 alone treatment. However, no obvious body weight loss happened during the experiments in nude mice treated with enzalutamide, IU1, or both (Fig. 7d). Subsequently, the level of AR, Ki67 and Bax were evaluated using immunochemical staining. We found that the expression of AR and Ki67, which promote proliferation, were decreased and Bax which promotes apoptosis was increased in the enzalutamide and IU1 combined treatment group (Fig. 7f). Apoptosis as indicated by TUNEL assays was increased in the combined treatment group as compared with either single drug treatment group (Fig. 7e). In separate cohort of mice, we implanted subcutaneously USP14-depleted MCF-7 cells stably expressing USP14-specific shRNA or control shRNA and monitored tumor growth as they were treated with enzalutamide or vehicle. Mice bearing USP14 shRNA-expressing MCF-7 cells showed decreased tumor growth compared with mice implanted with MCF-7 cells expressing control shRNA (Fig. 8a-c). More importantly, oral administration of enzalutamide exerted more effective tumor suppression in the USP14 shRNA group than in the control shRNA group as revealed by changes in the tumor volumes and weights whereas no difference in mouse body weight was observed among all groups (Fig. 8a-e). Immunochemical staining assay showed that AR, p-AKT and Ki67 expression were decreased and Bax expression were increased in the enzalutamide and USP14 shRNA combined group compared with the signal treatment (Fig. 8f).

## Discussion

Enzalutamide, a second-generation AR antagonist, has been approved by the U.S. Food and Drug Administration for therapy in the patients with castration-resistant prostate cancer (CRPC) [41, 42]. Compared with the first-generation AR inhibitors, such as bicalutamide and flutamide, enzalutamide shows a higher anticancer potency resulting from better affinity to AR and inhibition of AR nuclear translocation [43–45]. Enzalutamide reduces the mortality of men with metastatic CRPC by 37% and prolongs the overall survival [45]. Increasing studies have demonstrated that enzalutamide is a good treatment strategy for triple negative breast cancer (TNBC) as well as CRPC similarly through targeting AR [46, 47]. Clinical trials showed an anticancer effect of enzalutamide on patients with breast cancer, no matter it was combined with other chemotherapy or not [48].

Besides ER, PR and HER2, which promote the growth and progression of breast cancers, AR also may lead to the development of most breast cancers. AR has been identified as a potential therapeutic target in breast cancer, especially in the triple-negative breast cancer (TNBC) which shows the worst prognosis and metastases [49]. Studies have shown that ubiquitination regulates AR protein stability [21, 22]. Indeed, several DUBs have been explored to regulate AR protein levels and transactivation activity. USP7 modulates AR’s chromatin biding and thereby regulates AR activity and USP12 not only stabilizes AR proteins but also promotes AR transactivation activity by weakening ubiquitin-dependent degradation [50, 51]. Our previous studies has shown that USP14 can stabilize AR protein expression by trimming K48-linke ubiquitin chain on AR. Silencing or inhibiting USP14 triggers cell growth inhibition and cell cycle arrest by decreasing AR level in AR-positive breast cancer cells [21, 22]. However, no prior reported study has investigated whether USP14 inhibition in combination with AR antagonization has a benefit in treating breast cancer.

In the present study, we evaluated the effects of enzalutamide combined with USP14 inhibitor IU1 or with USP14 siRNA on breast cancer in vitro and in vivo. We have demonstrated that enzalutamide and USP14 inhibition synergistically inhibit cell viability in breast cancer cells; this combined growth inhibition effect of the two agents is predominantly due to more effective induction of cell cycle arrest and cell apoptosis. Flow cytometry analysis showed increased percentage of cells at G0/G1 phase and elevated proportion of cell death in the cells treated with a combination of enzalutamide and IU1/USP14 siRNA, compared with those received the single agent treatment. In addition, enzalutamide promotes the decreases in proteins levels of CDK4, Cyclin D1 and CDK2 and the increase in P27 protein induced by IU1 or USP14 knockdown. The increases in the cleavage of caspase-3, − 8, − 9 and PARP, as well as downregulation of Bcl-2 expression and elevation of Bax expression were more pronounced in the combined treatment group than in the single agent treatment groups. These findings indicate that activation of caspase pathway and mitochondrial dysfunction contribute to the anticancer synergy between enzalutamide and USP14 inhibition.

Both enzalutamide and USP14 inhibition can suppress AR signaling but they use different mechanisms to do that. As an AR antagonist, enzalutamide suppresses AR signaling at multiple steps, including competing with androgen in AR binding, blocking AR nuclear translocation, and preventing AR from binding the chromatin; USP14 inhibition, however, is known to destabilize AR protein and thereby reduce AR levels, which is confirmed in breast cancer cells by the present study. Therefore, we predicted that a treatment combining enzalutamide and USP14 inhibition should be more effective than the treatment with either single agent in terms of suppressing AR signaling in AR-positive breast cancer cells. Indeed, this prediction is well supported by our data. Enzalutamide alone did not cause a remarkable change in AR protein levels but USP14 inhibition alone or in combination with enzalutamide did. Furthermore, signaling events down stream of AR, such as the expression of IGF-1R,EGFR, and β-catenin, as well as GSK-3β and AKT phosphorylation, were more discernibly suppressed by the combination treatment than the single agent treatment (Fig. 5).

As an important non-stoichiometric DUB subunit of the 19S proteasome, USP14 has essential functions to the cell beyond AR regulation [31, 52]. Hence, the synergy between enzalutamide and USP14 inhibition in antitumor effect on breast cancer also may be attributable to additional tumor suppressing actions from USP14 inhibition, beyond suppressing AR signaling. This is underscored by the results from the AKT inhibition experiment (Fig. 6). AKT inhibition with a small molecule inhibitor (MK2206) alone induced growth inhibition and cell death, to the extent comparable to the treatment combining enzalutamide and USP14 inhibition; however, the treatment combining all three (i.e., enzalutamide + USP14 inhibition + AKT inhibition) showed significant greater effects than the former two treatment regimen (Fig. 6). Hence, it is very likely that suppressing the AR-PI3K/AKT signaling pathway participates in the synergy in the antiproliferation and proapoptosis between enzalutamide and USP14 inhibition while other mechanisms from USP14 inhibition also come into play. Importantly, the synergy in in the cytostatic effect on breast cancer cells between enzalutamide and IU1 or USP14 knockdown observed in cell cultures has been confirmed by our mouse xenograft experiments. Enzalutamide in combination with either pharmacological inhibition of USP14 with IU1 or shRNA-mediated USP14 knockdown were able to more effectively reduce tumor volumes and tumor weights than treatment with enzalutamide or USP14 inhibition alone, without discernibly altering mouse body weights, which also suggests that the two drug regimen is safe and well-tolerated.

## Conclusion

In summary, this study demonstrates a potential advantage of enzalutamide in combination of USP14 inhibition in AR-positive breast cancer treatment, compared with the treatment with each alone. By synergistically targeting AR-dependent and –independent pathways, this new combination treatment strategy may provide a potentially novel regimen for the treatment of AR-positive breast cancer in humans.

## Additional file

Additional file 1: **Figure S1.** Enzalutamide induced-antiproliferation is regulated by silencing AR or overexpressing USP14. (DOCX 422 kb)

## Abbreviations

AR: Androgen receptor
BCa: Breast cancer
CRPC: castration-resistant prostate cancer
DUBs: deubiquitinating enzymes
ER: estrogen receptor
HER2: human epidermal growth factor receptor 2
IGF-1R: IGF-1 receptor
PR: progesterone receptor
TNBC: triple-negative breast cancer
