# Supplementary material for: Inhibition of USP14 enhances the sensitivity of breast cancer to enzalutamide
Source: J Exp Clin Cancer Res. 2019 May 24;38:220. doi: 10.1186/s13046-019-1227-7 (PMC6534920; doi:10.1186/s13046-019-1227-7)
Supplement: Supplementary file 1 — Figure S1. Enzalutamide induced-antiproliferation is regulated by silencing AR or overexpressing USP14. (DOCX 422 kb) [file 13046_2019_1227_MOESM1_ESM.docx]

**Supplementary Methods**

**Plasmids and transfection of cells**

The plasmid MYC-USP14 encoding a fusion protein of USP14 and control vector was obtained from Genechem (Shanghai, China). MDA-MB453 cells were plated into 96-well and 6 cm plates for 24 h. The cells were transfected with plasmid MYC-USP14 or control vector mixed with lipofectamine 3000 transfection reagent (Invitrogen). After 6 h, fresh medium was replaced. Cells were incubated for addition 42 h.


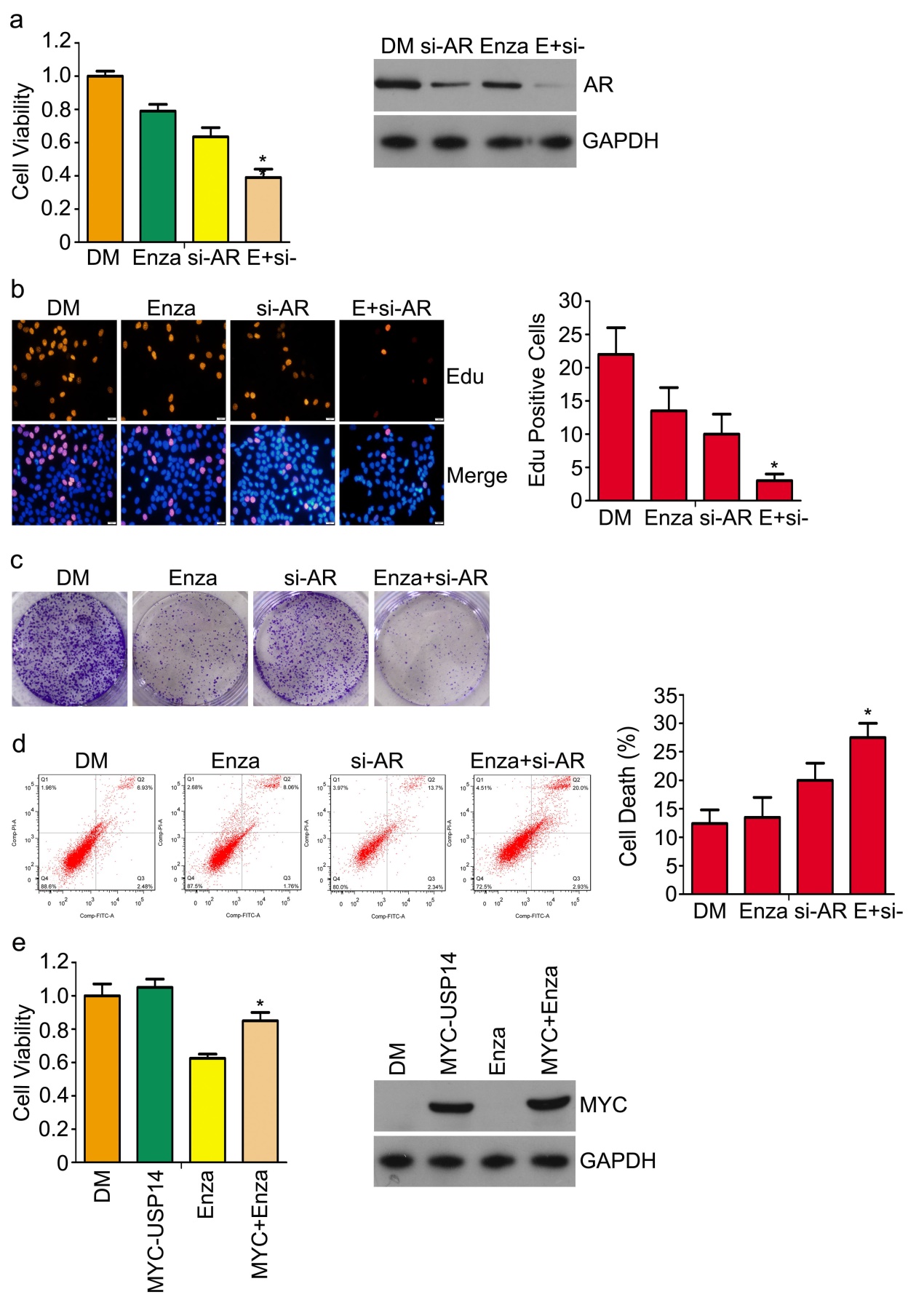


**Fig. S1. Enzalutamide induced-antiproliferation is regulated by silencing AR or overexpressing USP14.** (**a**) MDA-MB453 cells were transfected with AR siRNA (50 nM) and then treated with enzalutamide (20 μM) for 48 h. 20 μl MTS was incubated for 2 or 3 h. Cell viability was tested. The expression of AR protein is shown. GAPDH was a loading control. *p<0.05 *vs.* other treatment group. (**b**) Cells were treated with AR siRNA and enzalutamide for the indicated time and then were subjected to Edu staining. Represent images are shown and stained cells were calculated. The experiments are from three independent experiments. Scale bar, 50 μm. *p<0.05 *vs.* other treatment group. (**c**) Colony formation assay was performed in treated cells. Represent images are shown. (**d**) The indicated cells with AR siRNA or enzalutamide or the both were subjected to cells death assay. The showed images were from three independent experiments and cell death population were shown. *p<0.05 *vs.* other treatment group. (**e**) MDA-MB453 cells were transfected with MYC-tagged USP14 and then treated with enzalutamide for 72 h. Cells were incubated with 20 μl MTS and then cell viability was tested. *p<0.05 *vs.* enzalutamide treatment group.
